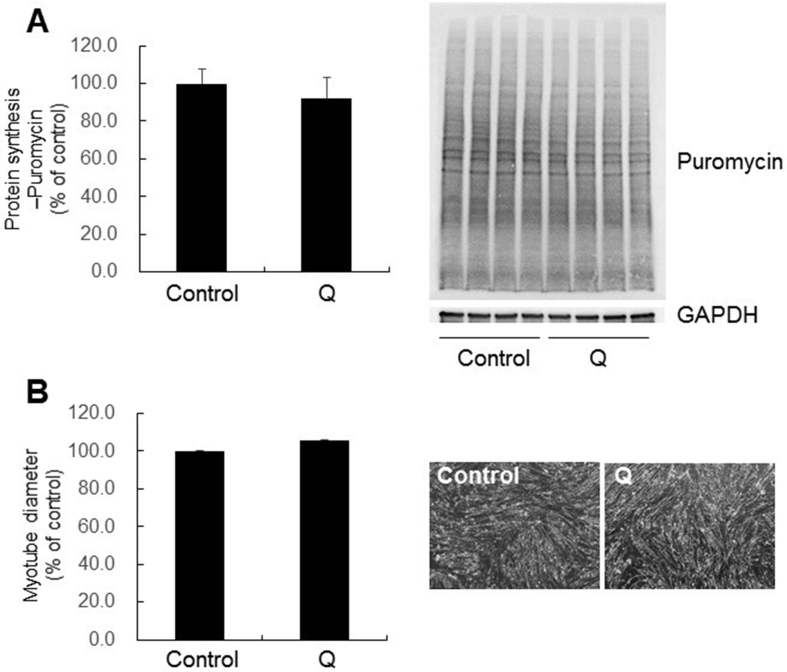# Quercetin glycosides prevent dexamethasone-induced muscle atrophy in mice

**DOI:** 10.1016/j.bbrep.2019.100618

**Published:** 2019-02-11

**Authors:** Yuta Otsuka, Kahori Egawa, Noriyuki Kanzaki, Takayuki Izumo, Tomohiro Rogi, Hiroshi Shibata

**Affiliations:** Institute for Health Care Science, Suntory Wellness Ltd, 8-1-1 Seikadai, Seika-cho, Soraku-gun, Kyoto, 619-0284, Japan

**Keywords:** Quercetin glycosides, Muscle atrophy, Myostatin, Dexamethasone, QGs, quercetin glycosides, DEX, dexamethasone, atrogin-1, atrogin-1/muscle atrophy F-box, MuRF-1, muscle ring finger protein 1, Foxo1, forkhead box O1

## Abstract

Although quercetin has numerous biological benefits, including preventing muscle atrophy due to disuse, no reports have been published to date about the preventive effects and molecular mechanisms underlying drug-induced muscle atrophy. Highly soluble and bioavailable quercetin glycosides (QGs) were used to examine the inhibition of dexamethasone (DEX)-induced muscle atrophy in vivo. Male BALB/cCrSlc mice were treated with or without QGs for 7 days ad libitum, followed by addition of DEX to their drinking water for a further 7 days. The weight of gastrocnemius (GM) adjusted by body weight was significantly decreased on day 7 after DEX treatment. DEX-induced decrease of GM weight was improved by QG co-administration on day 7. The mRNA levels of muscle atrophy-related genes in the gastrocnemius were significantly lowered by QGs on day 1. In particular, the expression of myostatin, a master regulator of muscle mass homeostasis, was suppressed to that of the control level. In murine C2C12 myotubes, quercetin elevated the phosphorylation of Akt, which are downstream of the myostatin pathway, as well as expression of atrogenes. We demonstrated the protective effect of QGs in DEX-induced muscle atrophy, which might depend on the suppression of myostatin signaling.

## Highlights

● Quercetin glycosides (QGs) prevented the DEX-induced loss of muscle weight in BALB/cCrSlc mice
● Muscle atrophy-related genes expression, especially myostatin, were suppressed by QGs administration
● Quercetin elevated the phosphorylation of Akt, which are downstream of the myostatin pathway to atrogenes, in C2C12 myotubes

## 1 Introduction

Skeletal muscle is an abundant tissue in the human body. It is involved not only in mobility and movement but also in glucose and lipid metabolism. Muscle atrophy is the loss of skeletal muscle mass as a consequence of increased myofibrillar protein degradation and its decreased synthesis. It occurs under various conditions such as fasting, disuse, injury, side effects of pharmaceutical therapy and aging. Muscle atrophy causes falls and therefore it has become a serious social problem, particularly in an aging society [1].

Glucocorticoids are widely used as therapeutic agents for diseases associated with inflammation. Although glucocorticoids are useful, high doses or long-term usage has serious side effects such as hyperglycemia, adrenal gland dysfunction, osteoporosis and muscle atrophy. A synthetic glucocorticoid, dexamethasone (DEX), induces muscle atrophy, which involves both induction of protein degradation and suppression of general protein synthesis [2]. Recent studies show that two muscle-specific E3 ubiquitin ligases, atrogin-1/muscle atrophy F-box (atrogin-1) and muscle ring finger protein 1 (MuRF-1), the so-called atrogenes [3], play key roles in muscle atrophy caused by immobilization and denervation [4,5], as well as DEX treatment [6]. Furthermore, their gene expression and activities are regulated by transcription factors from the class O type forkhead (Foxo) family [[4], [5], [6], [7]].

Muscle mass is also regulated by cytokines such as myostatin. Myostatin is a member of the TGF-β superfamily and a known negative regulator of muscle cell growth [8]. Myostatin binds to its receptor, activates Smad2 and dephosphorylates Akt [8]. These signals downregulate proliferation and differentiation of satellite cells and muscle hypertrophy [9]. Glucocorticoids stimulate the production of myostatin [10]. In myostatin knockout mice, both denervation [11] and immobilization [12] induce muscle atrophy, but DEX treatment does not [13], indicating that myostatin plays an important role in DEX-induced muscle atrophy.

Quercetin is a flavonoid widely distributed in edible plants such as onions and apples. There are many reports of its biological benefits including its antioxidative [14], anti-inflammatory [15], anti-obese [16] and oxygen radical-scavenging activities [17]. Recently, Mukai et al. [18,19] reported that quercetin prevents both disused and denervated muscle atrophy because of its antioxidant activity, although the effects of quercetin on DEX-induced muscle atrophy remains unknown.

In the present study, we treated mice with quercetin glycosides (QGs). QGs are more water soluble and bioavailable than quercetin aglycone, which does not exist in a glycoside or conjugate form [20]. When absorbed, QGs are enzymatically converted into the aglycone form and are found to have beneficial effects similar to those for the corresponding quercetin aglycone [20]. Thus, we aimed to investigate the protective effects of QGs in DEX-induced muscle atrophy.

## 2 Materials and methods

### 2.1 Chemicals

QGs were enzymatically manufactured at San-Ei-Gen F.F.I. (Osaka, Japan) from isoquercitrin prepared from quercetin-3-O-rutinoside and sophorin, as described previously [16]. Quercetin and DEX were purchased from Nacalai Tesque (Kyoto, Japan).

### 2.2 Animals

Seven-week-old male BALB/cCrSlc mice were purchased from Japan SLC Inc. (Shizuoka, Japan). The animals were housed in polycarbonate cages with wood pulp bedding, which were changed once a week. Mice were fed a pellet rodent diet (CE-2, CLEA Japan, Inc., Tokyo, Japan) and tap water. The facility was maintained under specific pathogen-free conditions at a temperature of 23 ± 2 °C and a humidity of 55 ± 10%, with a 12-h light/dark cycle. Before the experiment, the animals were acclimated to the facility for 1 week. After a 1-week acclimation, 0.15% or 0.45% w/v QGs were provided in the drinking water for 7 days (day −6 to day 0). Then, 0.001% w/v DEX was provided in the drinking water in addition to the QGs for a further 7 days (day 1 to day 7). Thus, QGs were provided for 14 days (day −6 to day 7). A control group received DEX only from days 1–7. For gene expression analysis, mice were sacrificed on days 1, 3 and 7. At each time point, the mice were weighed and euthanized, and the gastrocnemius (GM) was dissected and weighed. The GM was frozen in liquid nitrogen and stored at −80 °C until use in quantitative real-time PCR analysis. All protocols for animal procedures were approved by the Ethics Committee of Animal Experiment in accordance with the Internal Regulations on Animal Experiments at Suntory, which are based on the Law for the Humane Treatment and Management of Animals (Law No. 105, 1 October 1973, as amended on 30 May 2014). These experiments were conducted from July 2013 to November 2013.

### 2.3 Cell culture

Murine myotube C2C12 cells were purchased from the American Type Culture Collection (Manassas, VA, USA). C2C12 cells were cultured in Dulbecco's modified Eagle's medium (DMEM; Sigma, St. Louis, MO, USA) supplemented with 10% fetal bovine serum (Hyclone, Waltham, MA, USA) until 100% confluent at 37 °C in humidified 5% CO_2_ atmosphere. After reaching confluency, the culture medium was changed to DMEM supplemented with 2% horse serum (Sigma) for 6 days for differentiation. All media were changed every 2–3 days. After they were fully differentiated into myotubes, cells were treated with 1–10 μM quercetin for 24 h. After quercetin treatment, 1 μM DEX was added to the media to induce atrophy for 4 h in combination with quercetin. Cells were harvested and then used for quantitative real-time PCR and western blotting analyses.

### 2.4 Measurements of myotube diameters

To analyze diameters, two pictures were taken per well and 30 biggest myotubes from each picture were measured using Image J (National Institutes of Health, Frederick, MD, USA).

### 2.5 Measurements of protein synthesis using puromycin

Myotubes were incubated with 1 μM puromycin (EMD Millipore, Temecula, CA, USA) for 30 min. Lycates were prepared with Pierce M-PER (Thermo Fisher Scientific, Waltham, MA, USA) and protein concentration was measured for each lysate using the Pierce BCA Protein Assay Kit (Thermo Fisher Scientific). Five micrograms of protein were separated in a 10% polyacylamide gel (TEFCO, Tokyo, Japan) under denaturing conditions until the dye-front was ∼1.5 cm from the bottom of the gel. Proteins were transferred to PVDF membranes (Bio-Rad Laboratories, Hercules, CA, USA), and then incubated in Blocking One (Nacalai) and incubated overnight with a monoclonal antibody to puromycin (1:5,000 dilution, clone 12D10; Millipore EMD) or GAPDH (1:10,000 dilution, ab181602; Abcam, London, England) diluted in Blocking One. Membranes were then incubated with secondary IgG mouse antibody (1:20,000 dilution, ab197767; Abcam) or IgG rabbit antibody (1:10,000 dilution, NA934; GE Healthcare, Chicago, IL, USA). The total lane density was analyzed using Fusion FX (Vilber Lourmat, Collégien, France). Protein expression was normalized to that for GAPDH (loading control).

### 2.6 Quantitative real-time PCR

Total RNA was isolated from tissues using ISOGEN (Nippon Gene, Tokyo, Japan) and purified using the RNeasy Mini kit (QIAGEN, Hilden, Germany) as previously described [21]. Total RNA was also isolated from cells using the RNeasy Mini kit in accordance with the manufacturer's instructions. Isolated RNA, whose integrity was determined using a bioanalyzer (Agilent Technologies, Santa Clara, CA, USA), was reverse-transcribed to cDNA using the High-Capacity cDNA Reverse Transcription Kit (Life Technologies, Carlsbad, CA, USA). To measure the expression of mRNA, real-time PCR was performed using the TaqMan gene expression assay (Life Technologies) on an ABI 7900 Real-Time system (Life Technologies). All primers and probes were purchased as TaqMan Gene Expression Assays: F-box protein 32 (atrogin-1, Mm00499523_m1), tripartite motif-containing 63 (MuRF-1, Mm01185221_m1), forkhead box O1 (Foxo1, Mm00490672_m1), myostatin (myostatin, Mm01254559_m1), myosin heavy chain 7 (Myh7, Mm00600555_m1), myosin heavy chain 2 (Myh2, Mm01332564_m1), myosin heavy chain 1 (Myh1, Mm01332489_m1) and myosin heavy chain 4 (Myh4, Mm01332541_m1). The relative expression levels of the genes in each sample were determined by the comparative Ct method. Gene expression levels for each gene were normalized to that for 18S ribosomal RNA (Hs99999901_s1) and expressed as fold change relative to that of the control group.

### 2.7 Western blotting

Cells were lysed in iced-cold Pierce M-PER buffer containing Halt Protease and Phosphatase Inhibitor Cocktail (Thermo Fisher Scientific). After protein concentration was measured, protein expression was determined using an automated western blotting system according to the manufacturer's instructions (ProteinSimple, San Jose, CA, USA). Following capillary-based electrophoresis, primary antibodies against phosphorylated-Smad2 (p-Smad2, 1:50 dilution, #8828S; Cell Signaling Technology, Danvers, MA, USA), Smad2 (1:200 dilution, #5339S; Cell Signaling Technology), phosphorylated-Foxo3a (p-Foxo3a, 1:10 dilution, #5538S; Cell Signaling Technology), Foxo3a (1:50 dilution, #2497S; Cell Signaling Technology), phosphorylated-Akt (p-Akt, 1:10 dilution, #9271S; Cell Signaling Technology), Akt (1:100 dilution, #9272S; Cell Signaling Technology) and GAPDH (1:200 dilution, ab181602; Abcam) were infused into capillaries to probe target proteins and were visualized using labeled secondary antibodies. Protein expression was quantified using Compass Software (ProteinSimple).

### 2.8 Statistical analysis

Data are shown as the mean ± SE. Statistical analysis was performed using one-way ANOVA with Dunnett's test. *P*-values below 0.05 were considered significant. All statistical analyses were carried out using IBM SPSS Statistics for Windows, Version 21.0 (IBM Corp., Armonk, NY, USA).

## 3 Results

### 3.1 Effects of QGs on DEX-induced muscle atrophy in mice

The effects of quercetin on DEX-induced muscle atrophy were evaluated in vivo. The weight of GM adjusted by body weight decreased significantly (p < 0.05) by day 7 after DEX treatment compared to that of the control (Fig. 1A), although the weights on days 1 and 3 were not changed (Fig. 1B). DEX-induced decrease of GM weight increased significantly (p < 0.05) after co-administration with 0.45% QG by day 7 (Fig. 1A), but the weights on days 1 and 3 were not changed (Fig. 1B). DEX treatment affected MyHC isoform expression in GM, increasing in the percentage of *MyHCⅡx* (*Myh1*) and decreasing in that of *MyHCⅡb* (*Myh4*), although QG co-administration did not alter (Supplementary Fig. 1).

**Fig. 1:**
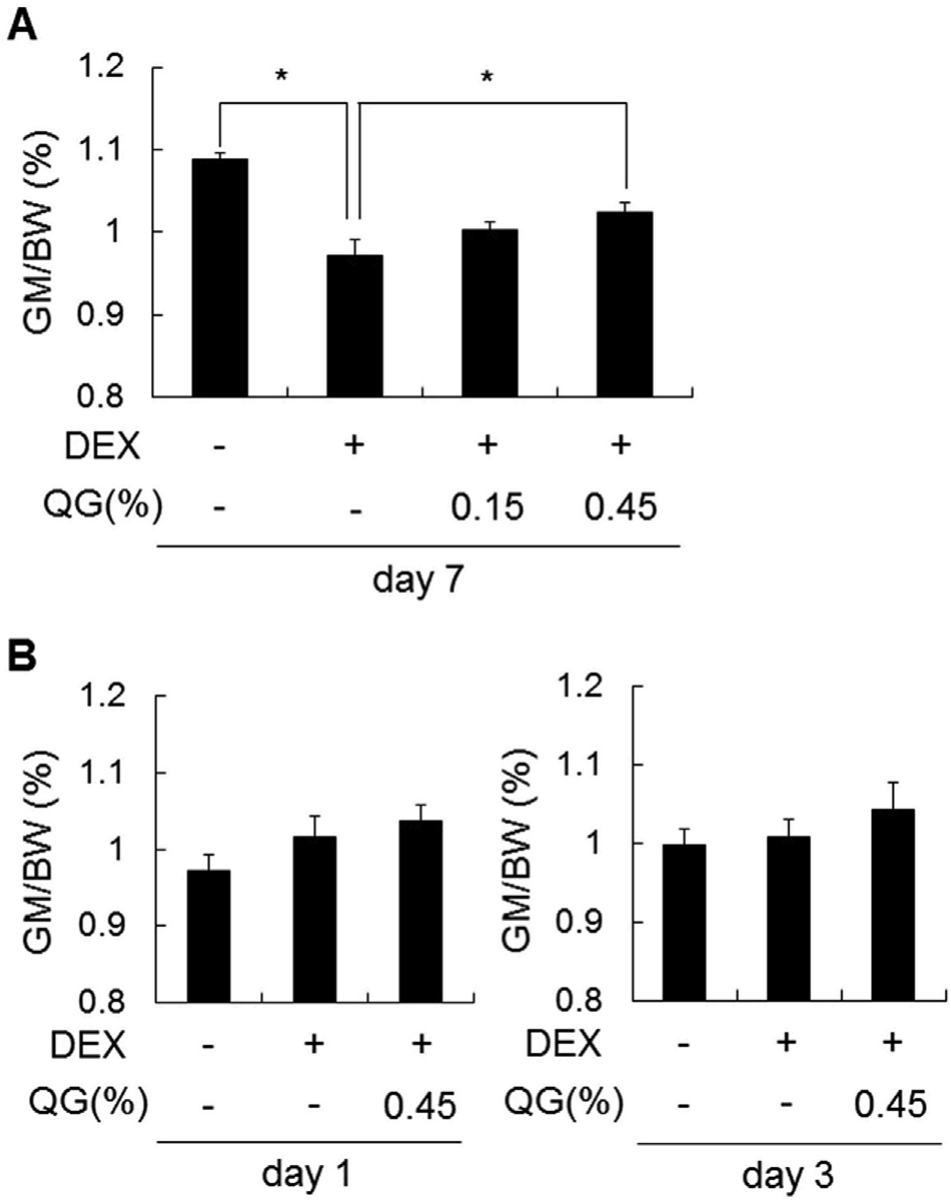
**Effects of QG administration on DEX-induced GM atrophy in BALB/cCrSlc mice**. Mice were administered 0.15% or 0.45% w/v QGs in drinking water for 7 days (day −6 to day 0) and then co-administered QGs with 0.001% w/v DEX (A) a further 7 days (day 7) or (B) 1 (day 1) or 3 days (day 3).Graphs express the weight of GM adjusted by body weight (BW). Values represent the mean ± SE (n = 8). Significant differences were determined by Dunnett's test (*p < 0.05).

### 3.2 Effects of QGs on muscle atrophy-related gene expression in mice

**Fig. 2:**
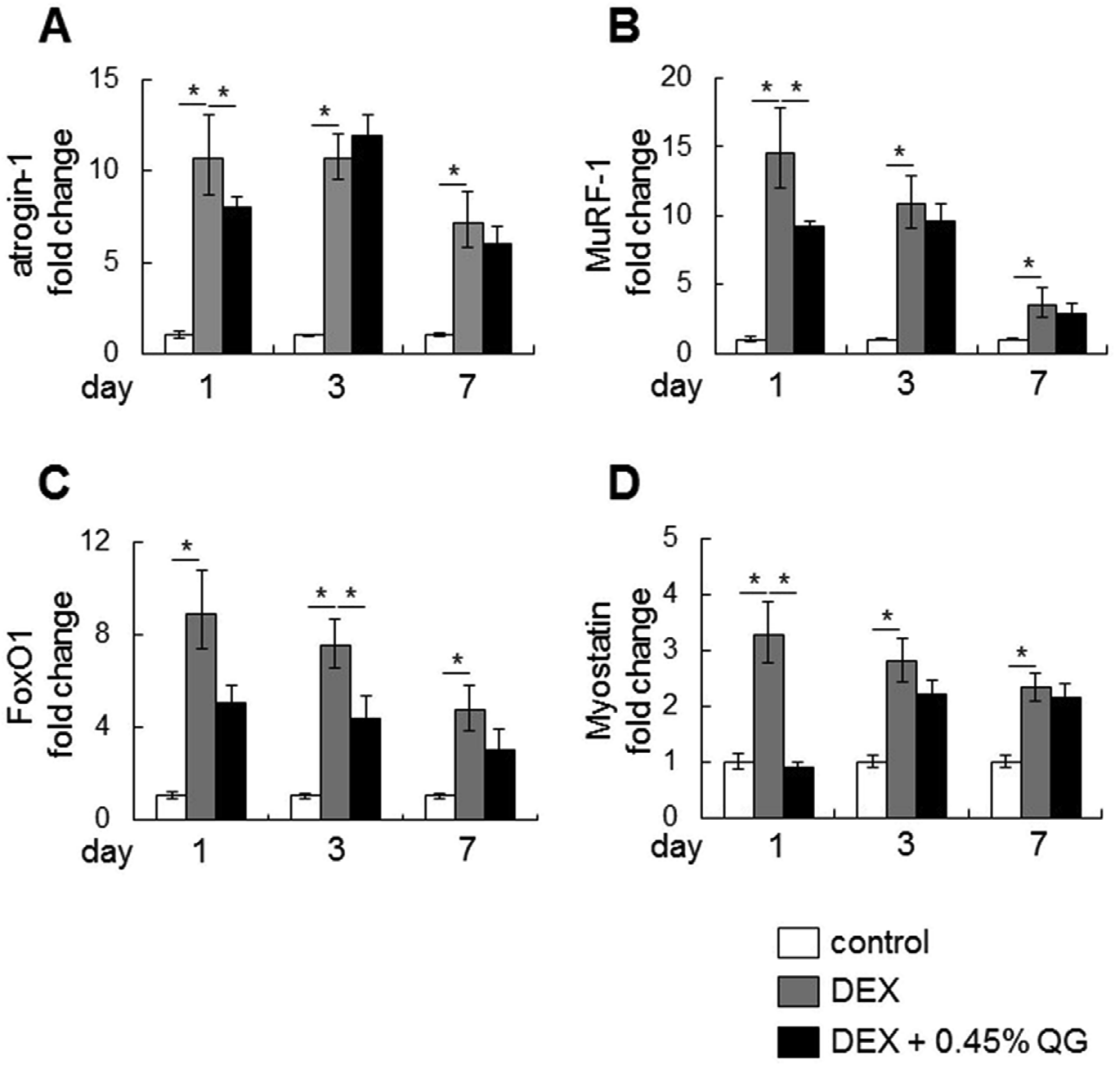
**Effects of QG administration on the expression of mRNAs related to muscle atrophy after 1-, 3- or 7-day DEX treatment**. Mice were administered 0.45% w/v QGs in drinking water for 7 days and then co-administered QGs with 0.001% w/v DEX for a further 1, 3 or 7 days (day 1, 3 and 7, respectively). Graphs express the relative gene expression of *atrogin-1* (A), *MuRF-1* (B), *Foxo1* (C) and *myostatin* (D). Values represent the mean ± SE (n = 5–8). Significant differences were determined by Dunnett's test (*p < 0.05).

The mRNA levels of genes related to muscle atrophy (*atrogin-1*, *MuRF-1*, *Foxo1* and *myostatin*) were increased by days 1, 3 and 7 after DEX treatment, although all mRNA levels decreased gradually over the period of DEX treatment (Fig. 2). DEX-induced mRNA levels of *atrogin-1*, *MuRF-1* and *myostatin* were significantly decreased (p < 0.05) after co-administration with 0.45% QG on day 1 (Fig. 2). In particular, *myostatin* expression was suppressed to that of the control level. *Foxo1* mRNA levels were also significantly decreased (p < 0.05) after co-administration with 0.45% QG by day 3 (Fig. 2).

### 3.3 Effects of quercetin on the phosphorylation signal related to muscle atrophy in C2C12 myotubes

**Fig. 3:**
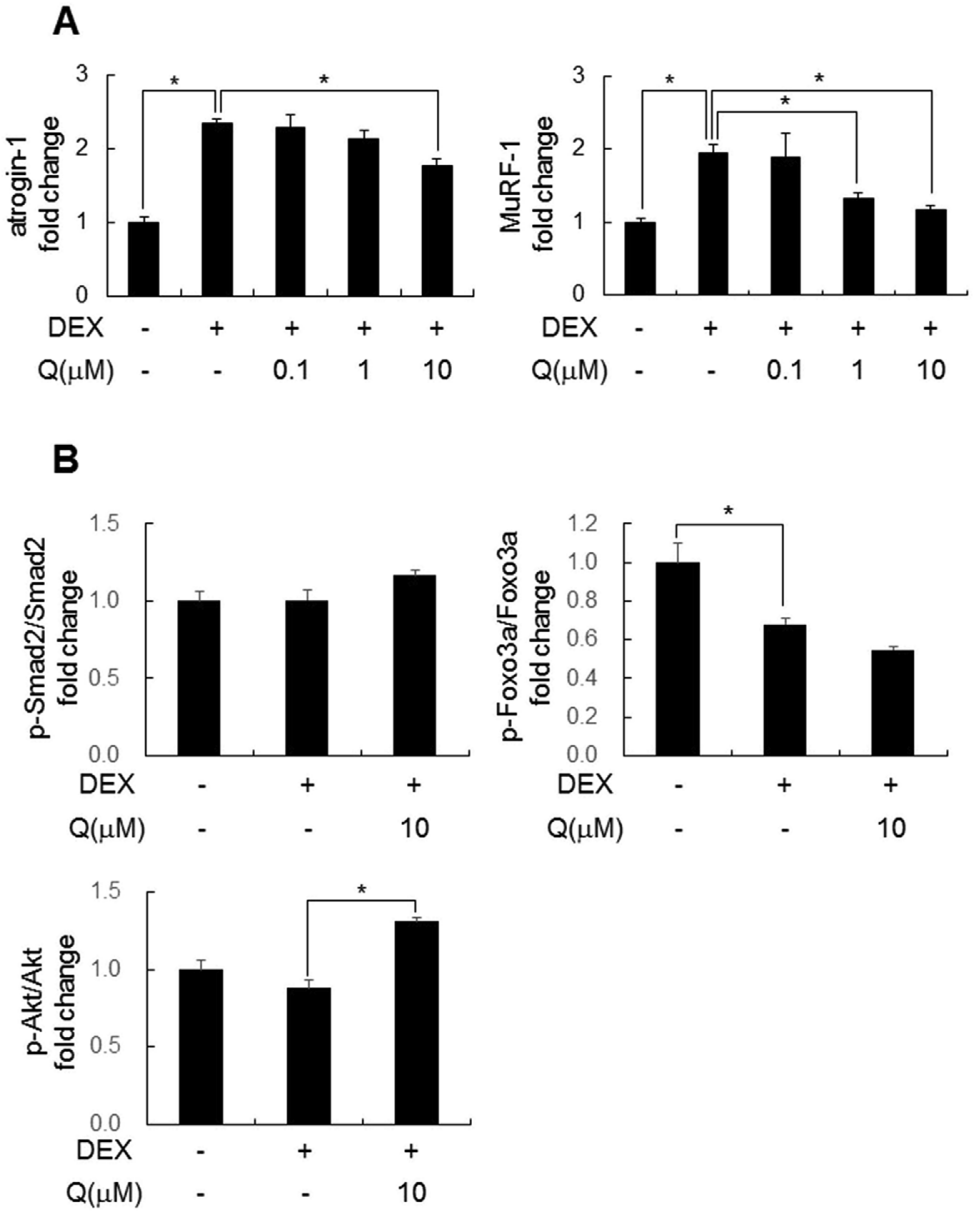
**Effects of quercetin on the phosphorylation signal related to muscle atrophy in C2C12 cells**. Cells were treated with DEX for 4 h after 24 h in the presence or absence of quercetin treatment. (A) Graphs express the relative gene expression of *atrogin-1* and *MuRF-1*. Values represent the mean ± SE (n = 5–6). (B) Graphs express the relative protein expression of p-Smad2/Smad2, p-Foxo3a/Foxo3a and p-Akt/Akt. Values represent the mean ± SE (n = 4). Significant differences were determined by Dunnett's test (*p < 0.05).

Next, we evaluated the effects of quercetin on the phosphorylation signal related to muscle atrophy, which is downstream of the myostatin pathway, using C2C12 myotubes. DEX-induced elevation of *atrogin-1* and *MuRF-*1 mRNA levels were suppressed with quercetin co-treatment in a concentration-dependent manner (Fig. 3A). The phosphorylation of smad2 and Foxo3a were not changed by co-treatment of quercetin, but those of Akt, which was slightly decreased by DEX alone, was significantly increased (p < 0.05, Fig. 3B).

We also evaluated the effects of quercetin on muscle hypertrophy. Neither muscle protein synthesis or muscle diameter was not changed by treatment of quercetin alone (Supplementary Fig. 2).

## 4 Discussion

In this study, we demonstrated that oral administration of QGs prevented DEX-induced muscle atrophy and expression of atrogenes in mice. The balance between myofibrillar protein degradation and synthesis determines muscle mass. DEX is well known to increase muscle degradation, suppress muscle synthesis and provoke muscle atrophy. Muscle proteolysis is caused by stimulation of the ubiquitin proteasome system, the lysosomal system and the calpain system. In the ubiquitin proteasome system, there are muscle-specific proteins called atrogenes, which play a critical role in DEX-induced muscle atrophy.

In our DEX-induced muscle atrophy model, the expression of *atrogin-1* and *MuRF-1* was highest on day 1 after DEX treatment. We used QGs in vivo because of the higher water solubility and bioavailability than quercetin aglycone. When orally administered, QGs are converted into quercetin aglycone, absorbed in the small intestine, and then distributed to various tissues in the aglycone form [20]. In C2C12 cells, we confirmed that quercetin suppressed the expression of *atrogin-1* and *MuRF-1* in a concentration-dependent manner. Contrary to our results, Hemdan DI et al. [22] reported that quercetin had no effects on DEX-induced atrogenes expression, which may be caused by higher dose of DEX than in our study and the previous report [23]. We also confirmed no effects of quercetin alone on muscle protein synthesis so that quercetin would have protective effects in the presence of atrophic-induced factors such as DEX.

In the presence of 0.45% QGs, a significant reduction in the expression of *atrogin-1* and *MuRF-1* was only observed on day 1. Sacheck et al. [4] also reported that expression of *atrogin-1 and MuRF-1* increased to a maximum level on day 3 after denervation, although the muscle weight did not differ from that for the control. Both results indicate that expression of atrogenes during the early phase of treatment is important in the process of muscle atrophy.

The balance between muscle proteolysis and protein synthesis is also regulated by myokines, cytokines secreted by the muscle itself. Myostatin is a myokine that plays an important role as a negative regulator in muscle hypertrophy [8]. In our study, co-administration of QGs completely inhibited the increase of myostatin expression by DEX treatment. Another report has described that the inhibition of myostatin in adult and older animals succeeds in increasing muscle mass [24]. Additionally, mutation of myostatin leads to increases in muscle mass in mice, sheep, cattle and humans [25]. Therefore, myostatin has attracted attention as a molecular target for suppressing the loss of muscle weight associated with aging and sarcopenia [26].

The myostatin gene promoter has a glucocorticoid response element motif. We confirmed that myostatin gene expression was increased by DEX treatment in vivo. Myostatin activates Smad2 signaling and downregulates Akt activation [8]. In C2C12 cells, recombinant myostatin protein reduced Akt phosphorylation and induced the expression of *atrogin-1*, which was abolished by Foxo1 knockdown using siRNA [27]. These data indicate that myostatin regulates atrogenes through the Akt-Foxo1 pathway. Corresponding to this regulatory pathway, we also observed the downregulation of *Foxo1* expression by QG administration. Additionally, myostatin knockout mice did not exhibit DEX-induced muscle atrophy and upregulation of atrogenes and *Foxo* expression [13]. Because myostatin functions as a master regulator of muscle mass homeostasis, the inhibition of myostatin could be the main contributor in the suppression of muscle atrophy by QGs.

Our results showed that quercetin elevated the phosphorylation of Akt in C2C12 myotubes. Corresponding to other reports, the phosphorylation of Foxo3a and Smad2, positive regulators for atrogenes, were not induced [28,29], although those of Akt, a negative regulator, was slightly decreased in this study rather than the previous report [23] because addition time of DEX was shorter. There results suggested that Akt signaling, which was up-regulated by quercetin, would mainly contribute to DEX-induced atrophic effects in the pathway from myostatin to atrogenes in C2C12 myotubes. Further studies were needed to clarify which factors were mainly involved in mechanism of anti-atrophic effects of quercetin.

In conclusion, we demonstrated that QGs prevented DEX-induced muscle atrophy. The downregulation of myostatin signaling via Akt phosphorylation may be one of the most important mechanisms responsible for the inhibitory effects of QGs on muscle atrophy.

## Funding

This research did not receive any specific grant from funding agencies in the public, commercial, or not-for-profit sectors.

## Conflict of interest

The authors have no conflicts of interest to declare.

## Figures and Tables

**figs1:**
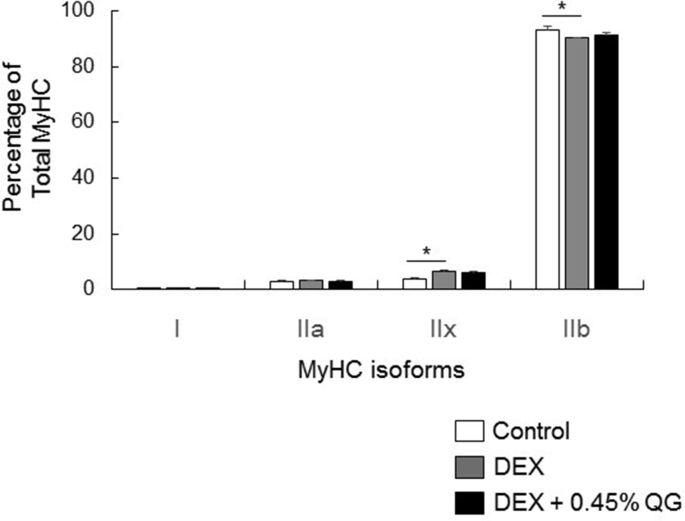


**figs2:**