# Supplementary material for: Quercetin glycosides prevent dexamethasone-induced muscle atrophy in mice
Source: Biochem Biophys Rep. 2019 Feb 11;18:100618. doi: 10.1016/j.bbrep.2019.100618 (PMC6372881; doi:10.1016/j.bbrep.2019.100618)
Supplement: Multimedia component 1 [file mmc1.docx]

**Supplementary Fig 1**

**Effects of QG administration on DEX-induced change of MyHC isoforms in GM.**

Mice were administered 0.45% w/v QGs in drinking water for 7 days (day −6 to day 0) and then co-administered QGs with 0.001% w/v DEX (A) a further 7 days. Graphs express the percentage of each MyHC isoform (*MyHCⅠ*, *MyHCⅡa*, *MyHCⅡx* and *MyHCⅡb*) to total MyHC gene expression. Values represent the mean ± SE (n = 8). Significant differences were determined by Dunnett’s test (*p < 0.05).

**Supplementary Fig 2**

**Effects of quercetin alone on muscle protein synthesis and myotube diameter in C2C12 cells.**

Cells were treated with quercetin for 24h. (A) Graphs express the relative muscle protein synthesis rate and the puromycin western blot form was indicated. (B) Graphs express the relative myotube diameter and the representative light microscopy images were indicated. Values represent the mean ± SE (n = 4).
